# Partial ulnar ostectomy, stereotactic body radiation therapy, and palliative radiation therapy as local limb sparing treatment modalities for ulnar tumors in dogs

**DOI:** 10.3389/fvets.2023.1172139

**Published:** 2023-07-12

**Authors:** Maureen A. Griffin, Tiffany Wormhoudt Martin, Douglas H. Thamm, Deanna R. Worley

**Affiliations:** ^1^Department of Clinical Sciences, Flint Animal Cancer Center, Colorado State University, Fort Collins, CO, United States; ^2^Department of Environmental and Radiological Health Sciences, Flint Animal Cancer Center, Colorado State University, Fort Collins, CO, United States

**Keywords:** neoplasia, osteosarcoma, radiation therapy, tumor, ulna, ulnectomy

## Abstract

**Background:**

Information on dogs that undergo limb preserving local treatment for ulnar tumors is currently limited.

**Objective:**

To describe the clinical characteristics and outcomes in dogs that underwent partial ulnectomy or radiation therapy (RT) for ulnar bone tumors, and to evaluate potential risk factors for outcomes as well as pre-treatment factors for association with treatment modality selected.

**Animals:**

Forty client-owned dogs that underwent partial ulnectomy or RT for an ulnar tumor from July 2006 to July 2021.

**Methods:**

The medical records database from a single institution were retrospectively reviewed, and data were recorded and analyzed.

**Results:**

Radiation therapy was performed in 24 dogs, with 12 stereotactic body RT (SBRT) and 12 palliative RT (PRT) plans, and partial ulnectomy was performed in 16 dogs. Biomechanical complications occurred in 6/12 (50%) dogs that underwent SBRT, 6/12 (50%) dogs that underwent PRT, and 3/16 (18.8%) dogs that underwent ulnectomy. The majority of dogs had a good functional outcome following partial ulnectomy, and no dogs required surgical stabilization of the carpus even with lateral styloid process excision. Pathologic fracture occurred in 4/12 (33.3%) dogs following SBRT and 5/12 (41.7%) dogs following PRT. Local progression or recurrence was documented in 5/12 (41.7%) dogs that underwent SBRT, 2/12 (16.7%) dogs that underwent PRT, and 2/16 (12.5%) dogs that underwent ulnectomy. The overall median survival time was 198 days, and factors that were significantly associated with improved survival time included adjuvant chemotherapy administration and partial ulnectomy as local treatment method for dogs that received chemotherapy.

**Clinical relevance:**

Both RT and ulnectomy were effective and well tolerated local treatment modalities for dogs with ulnar tumors.

## Introduction

The most common primary bone tumor in dogs is osteosarcoma, with a reported incidence of up to 87% of all skeletal neoplasms in this species (1–6). Overall, there is substantial literature regarding clinical presentation, treatment options, and outcomes for dogs with primary appendicular bone tumors. However, ulnar tumors have been uncommonly reported as an appendicular tumor site in dogs, and to date there are only two studies that specifically describe outcomes of dogs with ulnar osteosarcoma following a variety of treatment methods (7, 8). One retrospective study documented primary ulnar osteosarcoma in 12 dogs and reported a median survival time of approximately 8.5 months for treated dogs (7). In this study, 8 dogs were treated with partial ulnar ostectomy and 3 dogs were treated with full limb amputation (7). Radiation therapy (RT) was administered in 4 dogs prior to partial ulnar ostectomy; no dogs received RT alone for local treatment (7). In addition, 6 dogs in this study received chemotherapy (7). The largest retrospective study to date on ulnar osteosarcoma in dogs included 30 cases from 9 institutions (8). Partial ulnar ostectomy was performed in 11/30 dogs; only 3 dogs with partial ulnectomies had concurrent excision of the lateral styloid process (8). Full limb amputation was performed in 14/30 dogs, and chemotherapy was administered in 22/30 dogs (8). No dogs received RT as a primary treatment for local disease (8). The overall median survival time of dogs with ulnar osteosarcoma in this study was 463 days (8). With regards to primary local disease treatment with RT for canine ulnar tumors, a recent study on stereotactic body RT (SBRT) for appendicular osteosarcoma in 123 dogs included only 4 dogs that underwent radiation of the ulna alone, with survival ranging from 116 to 382 days in these 4 dogs (9).

Two additional studies have evaluated the effect of distal ulnar ostectomy with lateral styloid process excision (and concurrent disruption of the lateral collateral ligament) on carpal joint stability in cadaveric dogs (10, 11). One of these studies demonstrated slight increase in carpal valgus in a model mimicking weight bearing during stance, and the other study documented an increase in the carpal angle upon stress radiography (10, 11). However, as both of these studies were cadaveric in nature, clinical tolerance of distal ulnar ostectomy and these potential changes in carpal stability remain to be determined following partial ulnar ostectomy in live dogs.

Ultimately, information on the clinical findings, complications, and short-and long-term outcomes in dogs that undergo limb preserving partial ulnectomy or RT for ulnar tumors is currently limited. Our primary objectives were to describe the clinical characteristics as well as short-and long-term outcomes in dogs that underwent partial ulnar ostectomy or RT for ulnar bone tumors. We aimed to evaluate both disease progression outcomes and limb function outcomes in these dogs. Our secondary objectives were to evaluate potential risk factors for outcomes following treatment, as well as to evaluate any pre-treatment factors for association with local limb preserving treatment modality selected. We hypothesized that dogs undergoing partial ulnectomy or RT for treatment of ulnar tumors would have good long-term function of the limb without any need for additional surgical stabilization of the carpus, few overall complications, and overall survival times similar to previously reported for dogs with appendicular bone tumors.

## Materials and methods

The medical record database of the Colorado State University James L. Voss Veterinary Teaching Hospital was retrospectively searched to identify dogs that underwent partial ulnectomy or RT for primary treatment of an ulnar bone tumor. All dogs that had limb preserving local treatment performed at Colorado State University from July 2006 through July 2021 and had post-treatment follow-up information were included in the study. Information obtained from the medical records included signalment, history of orthopedic or neurologic disease, type and duration of clinical signs, physical examination findings, preoperative diagnostic results, surgical procedures and RT protocols performed, histopathologic results, neoadjuvant and adjuvant oncologic treatments, complications, progression of local and metastatic disease, post-treatment limb function and procedures performed for stabilization, and timing and cause of death or loss to follow-up.

Factors including signalment, history (including travel and trauma history), physical examination findings, lesion location and appearance on imaging, labwork results, staging diagnostics, and cytology/histopathology results (when available) were all taken into account in each patient’s diagnosis and treatment pursued. At our institution, pathologic fracture was considered exclusionary for RT but not ulnectomy, and overt tumor extension into the adjacent radius or proximal/distal joint or bone structures was considered exclusionary for ulnectomy but not RT. In addition, ideal candidates for partial ulnectomy were dogs with tumors in the mid-distal ulna, though proximal limits of ulnectomy extent have not been well characterized to date. Owners were informed of all feasible options and elected treatment modalities in line with their goals. Though offered in all cases, a cellular diagnosis was not required prior to treatment if ulnar neoplasia was strongly suspected on the basis of the aforementioned factors. Owners’ goals and elected treatment modality (palliative vs. definitive) were also considered with regards to importance of obtaining a definitive diagnosis prior to treatment. Imaging modalities for local tumor assessment as well as staging diagnostics were thoroughly discussed with each client, and modalities were chosen on the basis of specific client goals and findings for each patient; limb CT was required for dogs that underwent SBRT.

Lameness was graded as mild to severe, with mild lameness being difficult to observe or inconsistently observed, moderate lameness being consistently observed in some gaits but always weight bearing on the limb, and severe lameness being observed consistently at a walk and with minimal or no weight bearing on the limb. Complications were listed as grades 1–3 in accordance with the Veterinary Radiation Therapy Oncology Group criteria for acute (within 90 days of RT) and late (more than 90 days following RT) radiation-associated adverse events, as grades 1–4 in accordance with the CLASSIC (Classification for Intraoperative Complications) criteria for intraoperative complications and the Accordion criteria for postoperative complications, and as grades 1–5 in accordance with the Veterinary Cooperative Oncology Group – Common Terminology Criteria for Adverse Events (VCOG-CTCAE v2) for chemotherapy-associated adverse events (12–14). With regards to function of the limb following RT or ulnectomy, the following outcomes were characterized as biomechanical complications: non-weight bearing lameness on the limb, pathologic fracture of the ulna, osteomyelitis/infection associated with the ulnar tumor, significant apparent discomfort and self-trauma of the limb, implant failure, and instability of the carpal or elbow joint. When local tumor progression or recurrence was documented, this was based on histopathology/cytology results or progression of the ulnar mass grossly and/or on radiographs.

### Computed tomography

When a computed tomography (CT) scan was performed, this was done using either a Picker PQ2000 CT single slice helical scanner (before November 2009; Picker Medical Systems, Cleveland, OH), a Philips Gemini TF Big Bore 16-slice scanner (after November 2009 and before February 2020; Philips Medical Systems, Nederland, B.V.), or a Siemens Somaton Force 128-slice scanner (after February 2020; Siemens Medical Solutions, Malvern, PA). Dogs were positioned in lateral recumbency with the affected limb down. The majority of the body was placed into a moldable bag (Vac-Lock Cushions; CIVCO Medical Solutions, Coralville, IA) with the affected limb stretched away and secured using a thermoplastic net attached to an indexed carbon fiber board (CIVCO Medical Solutions, Coralville, IA). The board and moldable bags were indexed to the CT couch and, if the patient received radiation, the radiation couch as well.

Both non-contrast and contrast volumetric (helical) datasets were obtained through the affected limb and a portion of the body in the region. Omnipaque 350 (GE Healthcare, Princeton, NJ) contrast media was injected IV. Reconstructed images were created at 2.0 mm intervals using a 512 matrix and a smooth algorithm. A bone algorithm was also reconstructed from the pre-contrast series at 1.0 mm intervals.

### Nuclear scintigraphy

When nuclear scintigraphy was performed, approximately 0.30 millicurie/kg technetium Tc99m-labeled hydroxy-methylene diphosphonate was injected IV into a lateral saphenous vein. Static images were then performed of the entire skeleton 2 h following injection, and radiopharmaceutical uptake was evaluated.

### Positron emission tomography/CT

When positron emission tomography (PET)/CT was performed, approximately 0.15 millicurie/kg F-18 fluorodeoxyglucose was injected IV into a medial or lateral saphenous vein. At approximately 1 h post-injection, the patient was positioned in dorsal recumbency and a pre-and post-contrast CT of the entire body was obtained in 2 mm and 5 mm contiguous transverse images in a standard soft tissue algorithm. Immediately following the whole body CT, whole body PET scan was performed. When CT scan was performed concurrently for RT planning, the patient was initially positioned in lateral recumbency and RT planning CT of the limb was performed first as previously described, followed by repositioning into dorsal recumbency with administration of fluorodeoxyglucose for PET/CT scan.

### Radiation treatment planning

When a CT was available, both the pre-contrast and post-contrast CT scans were used for contouring and planning. Radiation treatment planning was performed using Varian Eclipse treatment planning system (Varian Medical Systems, Inc., Palo Alto, CA) for both inverse and forward treatment planning. Gross tumor volume (GTV) and organs at risk (OARs) were identified and contoured. A 2 mm internal expansion from the body contour was used to create the skin contour. A 0–2 cm clinical target volume (CTV) was extended proximally or distally from the GTV within the bone to include possible microscopic disease. A 3 mm planning target volume (PTV) expansion encompassed the CTV to account for daily positioning errors. The skin in all SBRT cases was considered an OAR. Target volumes were pulled out of contours that included OARs to meet normal tissue constraints for optimization for SBRT.

For dogs that received palliative radiation therapy (PRT) without a CT scan, a similar position to the CT scan was used without the moldable bag. Manual planning was performed and calculated with the assistance of a radiograph of the affected limb. Digital MV port films were performed to confirm accurate location of treatment. Similar to the computer-based plans, a 2 cm CTV was included along with a 1 cm region for PTV.

All plans were designed using planar or non-coplanar, isocentrically placed 6 or 10 MV radiation beams or 6 MV volumetric arc therapy (VMAT) for inverse and forward treatment planning. Radiation beams were modulated for SBRT plans using sliding-window technique with the intent of delivering 100% of the radiation prescription to 99% of the GTV and CTV and 95% of the PTV. For manual plans, 6 MV parallel opposed (equally weighted) portals were used for treatment.

### Partial ulnectomy procedure

All dogs that received partial ulnar ostectomy for primary treatment of their ulnar tumors underwent general anesthesia and a lateral approach to the ulna. Locations of the incisions, surgical dissection, and ostectomy locations varied on the basis of disease location and extent, though the ostectomy location was generally performed with margins of 1–3 cm of healthy bone relative to neoplastic tissue, and the dissection plane involved soft tissues beyond the tumor capsule so as not to penetrate the gross tumor. Carpal joint stability was assessed intraoperatively prior to closure for dogs that underwent lateral styloid process excision. Postoperative external coaptation was performed at the discretion of each surgeon.

### Statistical analysis

Demographic features of the groups were compared using Kruskal-Wallis tests or ANOVA depending on data normality for continuous variables and Bonferroni-corrected Chi-square tests for categorical variables, respectively. The overall survival time (OST) was defined as the interval between the date of first treatment and the date of death or last follow-up. The OST was estimated using the Kaplan–Meier method. Potential factors influencing OST were assessed using log rank analysis or Cox regression. All statistical tests were two-sided and the significance level was set at *p* < 0.05. Statistical analyses were performed using Prism 9 (GraphPad Software, San Diego, CA) and SPSS v26 (IBM, Armonk, NY).

## Results

Forty dogs met inclusion criteria. Pre-treatment data, including signalment, historical orthopedic or neurologic disease, clinical signs, physical examination findings, pertinent bloodwork results, thoracic limb imaging modalities, ulnar tumor cytology and histopathology results, staging diagnostic modalities, and known or suspected metastatic disease, can be found in Table 1. Radiation therapy was performed as the primary local treatment in 24 dogs, and partial ulnar ostectomy was performed as the primary local treatment in 16 dogs. Stereotactic and palliative RT protocols were performed in 12 dogs each. Tumor type, staging data, treatment modality, survival, and follow-up data for each dog is provided in Table 2.

**Table 1:** Pre-treatment data [either number (percent) of all dogs or median and range] including signalment, historical orthopedic or neurologic disease, clinical signs, physical examination findings, pertinent bloodwork results, thoracic limb imaging modalities, ulnar tumor cytology and histopathology results, staging diagnostic modalities, and known or suspected metastatic disease.

	Number (percent) of all dogs	Median	Range
Age (years)		9.4	1.4–13.2
*Sex*			
Male castrated	29 (72.5%)		
Female spayed	9 (22.5%)		
Male intact	2 (5.0%)		
*Breed*			
Golden retriever	7 (17.5%)		
Labrador retriever	6 (15.0%)		
Rottweiler	3 (7.5%)		
Mastiff	2 (5.0%)		
American Staffordshire	2 (5.0%)		
Great Dane	2 (5.0%)		
Bernese mountain dog	2 (5.0%)		
Anatolian shepherd	2 (5.0%)		
Mixed/other	14 (35.0%)		
*Historical data*			
History of orthopedic/neurologic disease	24 (60.0%)		
Prior medical/surgical management for orthopedic/neurologic disease	15 (37.5%)		
*Clinical signs*			
Duration of clinical signs prior to presentation to referral hospital (days)		30	4–307
Thoracic limb lameness	35 (87.5%)		
Apparent pain	29 (72.5%)		
Swelling or mass effect of antebrachium	32 (80.0%)		
Non-specific signs (hyporexia, lethargy, and/or weight loss)	6 (15.0%)		
*Physical examination*			
Vital parameters within normal limits	40 (100.0%)		
Weight		40	23.6–82
Body condition score (/9)^†^		5.5	4–9
Lameness	31 (77.5%)		
Mild lameness	13 (32.5%)		
Moderate lameness	5 (12.5%)		
Severe lameness	4 (10.0%)		
Lameness reported but not described	9 (22.5%)		
Palpable swelling or mass effect of antebrachium	30 (75.0%)		
*Bloodwork*			
Monocyte count (/uL)		435	0–1,200
Alkaline phosphatase (IU/L)		65	17–722
*Thoracic limb imaging*			
Radiographs	39 (97.5%)		
Computed tomography	23 (57.5%)		
*Cytology of ulnar tumor prior to treatment* ^‡^			
Osteosarcoma/sarcoma	17 (42.5%)		
Inconclusive	4 (10.0%)		
*Histopathology of ulnar tumor prior to treatment* ^§^			
Osteosarcoma	3 (7.5%)		
Chondrosarcoma	2 (5.0%)		
Sarcoma	1 (2.5%)		
Inconclusive	1 (2.5%)		
*Staging diagnostics*			
Thoracic radiographs	36 (90.0%)		
Thoracic computed tomography	8 (20.0%)		
Abdominal ultrasound	10 (25.0%)		
Nuclear scintigraphy	9 (22.5%)		
Positron emission tomography/computed tomography	6 (15.0%)		
Additional skeletal radiographs	12 (30.0%)		
*Known or suspected metastatic disease*	12 (30.0%)		

† Data available for 18/40 dogs.

‡ Performed in 21 dogs.

§ Performed in 7 dogs.

**Table 2:** Tumor type, stage at the time of treatment, treatment performed, survival, and follow-up data for each dog.

Dog	Tumor type (if available)	Known or suspected metastatic disease pre-treatment? (Y or N)	Local treatment modality: SBRT (stereotactic body radiation therapy), PRT (palliative radiation therapy), ulnectomy	Adjuvant chemotherapy protocols (agent and number of injectable administrations if known)	Post-local treatment survival or follow-up time (days)	Time at death (D) or last follow-up (LFU)
1	Osteosarcoma	N	SBRT	Carboplatin × 3	66	D
2	Osteosarcoma	N	SBRT	Carboplatin × 3	132	D
3		Y	PRT	Cyclophosphamide	134	D
4	Osteosarcoma	N	SBRT	Carboplatin × 2	158	D
5	Chondrosarcoma	Y	SBRT	Toceranib	199	D
6	Sarcoma	N	SBRT	Carboplatin × 3, doxorubicin × 1, cyclophosphamide, toceranib, masitinib	193	D
7	Osteosarcoma	N	SBRT	Carboplatin × 6	313	D
8		Y	PRT	N/A	132	D
9	Osteosarcoma	Y	PRT	N/A	108	D
10	Osteosarcoma	N	PRT	N/A	382	D
11	Osteosarcoma	N	SBRT	Carboplatin × 2	227	D
12		Y	SBRT	Carboplatin × 4	116	D
13		Y	PRT	Melphalan, doxorubicin	14	D
14		N	PRT	N/A	48	D
15		Y	SBRT	Carboplatin, doxorubicin, toceranib	200	D
16	Osteosarcoma	N	PRT	N/A	75	D
17	Chondrosarcoma	N	PRT	N/A	140	D
18	Osteosarcoma	Y	PRT	Toceranib, doxorubicin	109	D
19	Sarcoma	N	SBRT	Carboplatin	382	D
20		Y	PRT	Carboplatin	225	D
21		Y	PRT	Mitoxantrone, carboplatin, doxorubicin	148	LFU
22	Sarcoma	N	SBRT	Carboplatin × 6	320	D
23	Osteosarcoma	N	SBRT	Doxorubicin × 1, carboplatin × 3	117	D
24	Osteosarcoma	N	PRT	N/A	317	LFU
25	Osteosarcoma	N	Ulnectomy	Carboplatin × 3, doxorubicin × 3	1,045	D
26	Osteosarcoma	N	Ulnectomy	Carboplatin × 4	470	D
27	Osteosarcoma	N	Ulnectomy	Carboplatin × 2	63	D
28	Osteosarcoma	N	Ulnectomy	Carboplatin × 6, doxorubicin × 7 (30 mg/m^2^ × 4 and 10 mg/m^2^ × 3), lomustine × 2	626	D
29	Osteosarcoma	N	Ulnectomy	Carboplatin × 4	1,324	D
30	Osteosarcoma	N	Ulnectomy	Carboplatin × 6	392	D
31	Osteosarcoma	N	Ulnectomy	Carboplatin × 6	198	D
32	Osteosarcoma	N	Ulnectomy	Carboplatin × 4	267	D
33	Osteosarcoma	N	Ulnectomy	Carboplatin × 4	90	LFU
34	Osteosarcoma	N	Ulnectomy	Doxorubicin × 3	68	D
35	Osteosarcoma	N	Ulnectomy	N/A	86	D
36	Osteosarcoma	N	Ulnectomy	N/A	132	D
37	Osteosarcoma	Y	Ulnectomy	N/A	70	D
38	Osteosarcoma	N	Ulnectomy	Carboplatin × 4	204	D
39	Osteosarcoma	Y	Ulnectomy	Carboplatin × 2, toceranib	477	LFU
40	Osteosarcoma	N	Ulnectomy	Carboplatin × 2	66	LFU

### Stereotactic body radiation therapy

Of the 12 dogs that underwent SBRT, all had both limb radiographs and CT performed for accurate assessment of the bone lesion and disease extent. Ulnar tumor involvement was distal third in 6 (50%) dogs, middle third in 3 (25%) dogs, proximal third in 2 (16.7%) dogs, and proximal two-thirds in 1 (8.3%) dog. In addition, 2 dogs that underwent SBRT had imaging changes of the radius consistent with tumor invasion or local reaction, 1 dog had possible extension of disease into the elbow joint, and 1 dog had hypoattenuating foci of the humerus with differentials of benign change vs. metastatic lesions. At the time of treatment, 3/12 (25%) dogs that underwent SBRT had known or suspected metastatic disease. For dogs that underwent SBRT, the median total dose was 36 Gy (range 33–36) and all treatments were delivered over 3 fractions over a median of 3 days (range 3–7) duration. Various dose statistics can be found in Table 3. Histopathology was ultimately performed in 6 dogs that underwent SBRT as a primary local treatment; results were consistent with osteosarcoma in 4 dogs (*via* incisional biopsy in 2, excisional biopsy in 1, and necropsy in 1), chondrosarcoma in 1 dog (*via* incisional and excisional biopsy), and sarcoma (grade 3 soft tissue sarcoma or poorly productive osteosarcoma) in 1 dog (*via* incisional and excisional biopsy). Cellular diagnosis was obtained *via* cytology results alone in an additional 4 dogs that underwent SBRT; results were consistent with osteosarcoma in 3 dogs and sarcoma in 1 dog. All dogs survived to discharge after SBRT of the ulnar tumor. Following SBRT, 1 dog required external coaptation with a splint for 12 days, and 1 dog required external coaptation with a splint until euthanasia (158 days following RT) due to radial fracture with repair and subsequent pathologic fracture of both the radius and ulna.

**Table 3:** Median, mean, and ranges of doses (in Gy) for variables from all stereotactic body radiation therapy (SBRT) treatments performed in the study.

	98% Gross tumor volume (GTV)	98% Clinical target volume (CTV)^†^	98% Planning target volume (PTV)	Conformity index	Gradient index
Median	29.8	27.7	19.8	0.86	0.85
Mean	27.9	27.5	19.0	0.81	0.89
Range	17.4–36.9	18.1–35.7	4.4–33.4	0.59–1.02	0.68–1.13

† Data available for 6/12 dogs.

During the course of SBRT, 1 dog developed an ipsilateral radial fracture prior to the second treatment (this dog had pre-RT imaging changes of the radius consistent with potential tumor invasion), and 1 dog developed aspiration pneumonia. Of the 12 dogs that underwent SBRT, 6 (50%) developed acute RT-associated complications, with 1 dog experiencing 2 complications: 2 grade 1, 2 grade 2, and 3 grade 3. All acute RT complications were associated with the skin. However, of the dogs with grade 3 acute complications, one underwent radial fracture repair during the course of SBRT (such that the skin complication may be attributed to the surgical procedure and/or SBRT), and one had an ulcerated lesion prior to RT that healed but developed additional regions of abscessation following SBRT. In addition, 4/12 (33.3%) dogs that underwent SBRT developed late RT-associated complications: 1 grade 1 (bone), 1 grade 2 (skin), and 2 grade 3 (skin) with one of the grade 3 complications resulting in death (this dog developed skin ulceration and an open wound over the tumor, and the owner elected euthanasia). Overall, 6/12 (50%) dogs that underwent SBRT experienced biomechanical complications following RT. Pathologic fracture was diagnosed in 4/12 (33.3%) dogs that underwent SBRT at a median of 117 days (range 55–301) following RT completion. Local tumor progression was documented in 5/12 (41.7%) dogs that underwent SBRT at a median of 137 days (range 95–308) following RT. Two dogs had both local tumor progression and pathologic fracture documented post-SBRT.

Following SBRT, 4/12 (33.3%) dogs underwent surgery on the irradiated limb: limb amputation in 2/12 (16.7%) dogs, partial ulnectomy in 1/12 (8.3%) dog, and ipsilateral radial fracture repair in 1 (8.3%) dog. No dogs that underwent SBRT received an additional course of RT.

### Palliative radiation therapy

Of the 12 dogs that underwent PRT, 11 (91.7%) had limb radiographs and 4 (33.3%) had limb CT performed for assessment of the ulnar tumor. Ulnar tumor involvement was distal third in 3 (25%) dogs, middle third in 4 (33.3%) dogs, proximal third in 1 (8.3%) dog, and not specified, predominantly due to poor resolution of port films, in 4 (33.3%) dogs. In addition, 2 dogs that underwent PRT had imaging changes of the radius consistent with tumor invasion or local reaction, and 1 dog had polyostotic lysis of the proximal ulna and radius as well as distal humerus (histopathology of this dog’s tumor, obtained from the humeral lesion, was consistent with chondrosarcoma). At the time of treatment, 7/12 (58.3%) dogs that underwent PRT had known or suspected metastatic disease. For dogs that underwent PRT, the median total dose was 16 Gy (range 10–20) administered over a median of 2 fractions (range 1–4) and a median of 2 days (range 1–10) duration with a median of 8 Gy/fraction (range 8–12). Histopathology was ultimately performed in 4 dogs that underwent PRT as a primary local treatment; results were consistent with osteosarcoma in 2 dogs (*via* excisional biopsy), chondrosarcoma in 1 dog (*via* incisional biopsy), and inconclusive in 1 dog (*via* incisional biopsy). Cellular diagnosis was obtained *via* cytology results alone in an additional 3 dogs that underwent PRT; results were consistent with osteosarcoma in all 3 dogs. All dogs survived to discharge after PRT of the ulnar tumor. No dogs required external coaptation following PRT.

Of the 12 dogs that received PRT, no dogs had any documented complications during RT or any acute or late RT-associated complications. Overall, 6/12 (50%) dogs that underwent PRT experienced biomechanical complications following RT. Pathologic fracture was diagnosed in 5/12 (41.7%) dogs that underwent PRT at a median of 74 days (range 27–219) following RT completion. Local tumor progression was documented in 2/12 (16.7%) dogs that underwent PRT at a median of 39 days (range 14–63) following RT. One dog had both local tumor progression and pathologic fracture documented post-PRT.

Following PRT, 2/12 (16.7%) dogs underwent surgery on the irradiated limb: both of these dogs had limb amputation performed. In addition, 2/12 (16.7%) dogs underwent a second course of PRT 2 months following initial treatment: 8 Gy/fraction over 2 fractions for one dog, and 10 Gy over 1 fraction for the other dog.

### Partial ulnar ostectomy

Of the 16 dogs that underwent partial ulnar ostectomy, all had limb radiographs and 7 (43.8%) had limb CT performed for ulnar tumor assessment. Ulnar tumor involvement was distal third in 14 dogs and middle third in 13 dogs (with multiple dogs having overlapping regions of ulnar involvement); no dogs that underwent ulnectomy had proximal ulnar tumors. In addition, 5 dogs that underwent ulnectomy had imaging changes of the radius consistent with local reaction (though tumor invasion could not be ruled out), and no dogs had concurrent lesions within the humerus or elbow joint. Surgical planning was based on thoracic limb radiographs alone in 9/16 (56.3%) dogs and thoracic limb CT and radiographs in 7/16 (43.8%) dogs. Two (12.5%) dogs that underwent partial ulnectomy had known or suspected metastatic disease at the time of treatment. For dogs that underwent partial ulnar ostectomy as primary local treatment, a median of 9.8 cm (range 6–14.1) of ulnar bone was excised (reported in 13 dogs), and the approximate percentage of ulna excised ranged from 50 to 66% (reported in 6 dogs). The lateral styloid process was excised in 8/16 (50%) dogs, and the interosseous ligament was partially transected in 4/16 (25%) dogs and completely transected in 4/16 (25%) dogs. In one dog with an excised lateral styloid process, subjectively excessive carpal laxity was noted intraoperatively, and soft tissues were imbricated with suture to aid in joint stability; no concurrent carpal stabilization procedures were performed for any dog. In all 4 dogs that had complete transection of the interosseous ligament, additional surgical procedures were performed to stabilize the remaining ulna and radius; this was performed with hemicerclage wire in 3 dogs and 80 lb. nylon leader line with crimps in 1 dog, with all implants placed through a hole drilled in the ulna and around the radius. None of the 4 dogs with partial interosseous ligament transection underwent stabilization procedures. No dogs had residual gross disease reported at the time of surgery completion. The median time for the partial ulnar ostectomy procedure was 95 min (range 60–160). For all 16 dogs that underwent partial ulnectomy as a primary local treatment, histopathology of the tumor was consistent with osteosarcoma. Complete excision was reported in 12/16 (75%) dogs, incomplete excision was reported in 2/16 (12.5%) dogs, and margins of excision were not reported in 2/16 (12.5%) dogs.

All dogs survived to discharge following ulnar ostectomy, and all dogs were discharged 1 day postoperatively. Following surgery, external coaptation was documented in 15/16 (93.8%) dogs for a median of 14 days (range 3–37, reported in 11 dogs); coaptation included a temporary splint for stability support in 8 dogs and a soft padded bandage only for mild compression without stability in 7 dogs. Of the 8 dogs that underwent lateral styloid process excision, all had some form of external coaptation: splint use in 6/8 and soft padded bandage only in 2/8. Of the 4 dogs that underwent partial interosseous ligament transection, 2/4 had a splint placed and 2/4 had a soft padded bandage only. Of the 4 dogs that underwent complete interosseous ligament transection, 3/4 had a splint placed and 1/4 had a soft padded bandage only.

Intraoperative surgical/anesthetic complications occurred in 11 dogs, with several dogs experiencing multiple complications: 5 grade 1 (hypercapnia/hypothermia/hypotension in 3, tumor capsule rupture in 1, and ulna fracture prior to ostectomy without breakage of the tumor capsule in 1), 10 grade 2 (hypotension in 6, mild to moderate hemorrhage requiring hemoclips in 1, hypotension and mild to moderate hemorrhage requiring hemoclips in 1, bradycardia in 1, and excessive carpal laxity requiring suture imbrication in 1 as noted above). Postoperative complications occurred during hospitalization in 1 dog (grade 1 [tachycardia]) and within 30 days postoperatively in 3 dogs (2 grade 1 [transient carpal instability in 1, hemicerclage implant failure in 1], 1 grade 2 [swelling and pain of the surgical site]). No dogs experienced grade 3–4 complications associated with partial ulnectomy. Overall, 3/16 (18.8%) dogs experienced biomechanical complications following ulnectomy. This included breakage of the hemicerclage wire in 2 dogs at 12 and 33 days postoperatively, and transient carpal instability in 1 dog that was noted 4 days postoperatively and resolved after 2 weeks with temporary external coaptation (this was the same dog with carpal laxity noted intraoperatively). No dogs developed fractures following partial ulnectomy. Local tumor recurrence was documented in 2/16 (12.5%) dogs at a median of 62 days (range 58–66) postoperatively; one of these dogs had complete excision on histopathology, and the margins of excision were not reported for the other dog though intraoperative rupture of the tumor capsule occurred.

Fourteen dogs were re-evaluated at 10–18 days postoperatively: 5 dogs had no reported lameness, 6 dogs had mild lameness, 2 dogs had moderate lameness, and 1 dog had severe lameness. The most recent/last examination was performed at a median of 119 days (range 38–720) postoperatively for 12 dogs, and at this time 8 dogs had no reported lameness, 3 dogs had mild lameness, and 1 dog had moderate lameness. The dog with long-term moderate lameness had breakage of the hemicerclage wire with subsequent external coaptation for 35 days postoperatively; this dog never developed overt elbow instability or pain or crepitus on elbow range of motion.

No additional surgical treatment or RT was performed following partial ulnectomy in any dog.

### Adjuvant chemotherapy and metastatic disease

Overall, 30/40 (75%) dogs received adjuvant chemotherapy, with multiple protocols used. For dogs in which the date of initiation of chemotherapy was known, the chemotherapy was started at a median of 1.5 days (range 0–29) following initiation of RT and 15 days (range 10–21) following surgery. Protocols included carboplatin in 25 dogs, doxorubicin in 9 dogs, toceranib in 5 dogs, cyclophosphamide in 2 dogs, and melphalan, masitinib, mitoxantrone, and lomustine in 1 dog each. Of the 30 dogs that received chemotherapy, single agent protocols were utilized in 21/30 (70%) and multi-agent protocols were utilized in 9/30 (30%). One dog was treated initially with a metronomic chemotherapy protocol (cyclophosphamide administered every other day). For dogs that received carboplatin, a median of 4 doses (range 2–6) was administered with doses ranging from 240 to 300 mg/m^2^. Chemotherapy was administered to 13/16 (81.3%) dogs that underwent ulnectomy. All 12 dogs that underwent SBRT received adjuvant chemotherapy, and 5/12 (41.7%) dogs that underwent PRT received adjuvant chemotherapy. Following chemotherapy, 18 adverse events were reported in 12/30 (40%) dogs. Eight adverse events were grade 1, 6 adverse events were grade 2, and 4 adverse events were grade 3. Twelve adverse events were related to blood/bone marrow toxicity, and 6 adverse events were related to gastrointestinal toxicity.

Following primary local treatment with RT or ulnectomy, new onset of metastatic disease (i.e., not including the 12/40 [30%] dogs that had metastatic disease pre-treatment) was detected in 11 (27.5%) dogs at a median of 85 days (range 64–438) post-treatment (for a total of 23/40 [57.5%] dogs with suspected metastatic disease overall).

### Demographic data relative to treatment type

Multiple demographics were evaluated to assess for a difference in the population of dogs that received partial ulnar ostectomy, SBRT, or PRT as primary local limb sparing treatment for their ulnar tumors. There was no detected trend to significance in the median age (*p* = 0.67), median weight (*p* = 0.26), sex (*p*-value range 0.12–1.0), breed (*p*-value range 0.055–0.88), incidence of historical orthopedic or neurologic disease (*p* = 0.59), incidence of alkaline phosphatase (ALP) elevation (*p* = 0.38), or incidence of known or suspected metastatic disease (*p* = 0.36) between dogs that underwent ulnectomy, SBRT, or PRT. However, a trend to significance was detected relative to ulnar tumor location for mid (*p* = 0.0023) and distal (*p* = 0.028) ulnar tumors but not for proximal (*p* = 0.096) ulnar tumors. When each subgroup was evaluated, a significant difference was found for mid ulnar tumors between dogs that underwent ulnectomy vs. SBRT (*p* = 0.018, with more dogs with mid ulnar tumors undergoing surgery than SBRT) but not SBRT vs. PRT (*p* > 0.999) or ulnectomy vs. PRT (*p* = 0.057). In addition, a significant difference was found for distal ulnar tumors between dogs that underwent ulnectomy vs. PRT (*p* = 0.0045, with more dogs with distal ulnar tumors undergoing surgery than PRT) but not ulnectomy vs. SBRT (*p* = 0.13) or SBRT vs. PRT (*p* = 0.4).

### Incidence of local recurrence/progression, amputation, biomechanical complications, and pathologic fracture relative to treatment type

Incidence of local tumor progression or recurrence, amputation post-treatment, biomechanical complications, and pathologic fracture were compared between dogs that underwent ulnectomy, SBRT, and PRT. No trend to significant difference was found between primary local treatment groups for the incidence of local progression or recurrence (*p* = 0.682), subsequent amputation (*p* = 0.128), or biomechanical complications (*p* = 0.0768). A trend to significance was detected with regards to incidence of pathologic fracture (*p* = 0.0072). When each treatment subgroup was compared for incidence of pathologic fracture, a significant difference was found between dogs that underwent ulnectomy vs. PRT (*p* = 0.0243, with a significantly greater incidence of pathologic fracture for dogs treated with PRT than ulnectomy) but not between dogs that underwent ulnectomy vs. SBRT (*p* = 0.0726) or SBRT vs. PRT (*p* > 0.999).

### Outcomes for all treatment groups

At the time of study completion, 35 dogs were dead, 3 dogs were alive, and 2 dogs were lost to follow-up. The median OST for all dogs was 198 days (range 14–1,324). Of the dogs that were dead, all deaths were possibly attributed to ulnar tumor-associated disease (local or systemic). The median OST was 267 days (range 63–1,324) for dogs that underwent partial ulnar ostectomy, 196 days (range 66–382) for dogs that underwent SBRT, and 133 days (range 14–382) for dogs that underwent PRT as a primary local treatment modality. The median OST was 204 days (range 14–1,324) for all dogs that received adjuvant chemotherapy and 132 days (range 48–382) for all dogs that did not receive adjuvant chemotherapy. For the subpopulation of dogs that did receive chemotherapy, the median OST was 392 days (range 63–1,324) for dogs that underwent partial ulnar ostectomy, 193 days (range 66–382) for dogs that underwent SBRT, and 167 days (range 14–225) for dogs that underwent PRT.

A trend toward significance was detected when dogs receiving ulnectomy, SBRT, and PRT were compared relative to OST (*p* = 0.033). In addition, this trend toward significance based on treatment group (ulnectomy, SBRT, and PRT) was also detected for the subpopulation of dogs that received adjuvant chemotherapy (*p* = 0.019). However, the only significant difference that remained when each subgroup was analyzed against each other was a significantly longer survival time for dogs that underwent ulnectomy and adjuvant chemotherapy compared to dogs that underwent SBRT and adjuvant chemotherapy (*p* = 0.036); no other two subgroup comparisons were significantly different relative to survival (all dogs that underwent ulnectomy vs. SBRT [*p* = 0.201], all dogs that underwent ulnectomy vs. PRT [*p* = 0.279], all dogs that underwent SBRT vs. PRT [*p* = 0.999], dogs that underwent ulnectomy or PRT with adjuvant chemotherapy [*p* = 0.221], and dogs that underwent SBRT or PRT with adjuvant chemotherapy [*p* > 0.999]). When RT subgroups (SBRT and PRT) were combined, a significant difference was found in survival between dogs that underwent ulnectomy vs. RT (*p* = 0.029; HR 0.437, 95% CI 0.21–0.92) and between dogs that underwent ulnectomy with adjuvant chemotherapy vs. RT with adjuvant chemotherapy (*p* = 0.007; HR 0.275, 95% CI 0.11–0.70). Dogs that received any primary treatment with adjuvant chemotherapy had improved survival times compared to dogs with any primary treatment that did not receive adjuvant chemotherapy (*p* = 0.045; HR 0.41, 95% CI 0.17–0.98). Age (*p* = 0.536), sex (*p* = 0.89), weight (*p* = 0.138), ALP level (*p* = 0.355), monocyte count (*p* = 0.61), relative location of the tumor within the ulna (*p* = 0.74), and documented or suspected metastatic disease pre-treatment (*p* = 0.23) were not found to be significantly associated with OST in this population of dogs.

## Discussion

To date, information on clinical findings, complications, and both functional and disease progression outcomes of dogs undergoing partial ulnar ostectomy or RT for an ulnar bone tumor has been limited due to a lack of data. Consistent with the overall literature for dogs with primary bone tumors, this study found that administration of adjuvant chemotherapy was associated with improved survival times in dogs with ulnar tumors that underwent either surgery or RT relative to dogs that received local treatment without adjuvant chemotherapy. Interestingly, in addition in this population, a trend to significance was seen when all three local treatment groups (partial ulnectomy, SBRT, PRT) were compared with regards to survival time, both for all dogs in the study and for the subset of dogs that underwent adjuvant chemotherapy. Overall, we found survival times similar to previously reported for dogs with appendicular bone tumors.

The only demographic factor that was found to be significantly different between treatment groups was ulnar tumor location, and all dogs that underwent partial ulnectomy had tumors in the mid and/or distal ulna. Although no demographic difference was seen with regards to suspected metastatic disease and primary treatment type elected, the majority of dogs with metastatic disease received PRT (7/12, 58.3%) compared to SBRT (3/12, 25%) or surgery (2/16, 12.5%). In addition, a greater proportion of dogs that underwent SBRT (12/12, 100%) received adjuvant chemotherapy than those that underwent PRT (5/12, 41.7%). It is important to consider that additional factors, such as potential metastatic disease and larger tumor volumes or more extensive primary disease, may have contributed to differences in outcomes, and greater sample sizes would be required to detect these differences.

With regards to function, the incidence of biomechanical complications approached, but did not reach, significance relative to treatment type. However, it is interesting to note that biomechanical complications occurred in half of all dogs that underwent SBRT or PRT and the minority (18.8%) of dogs that underwent partial ulnectomy. No significant difference was detected in the incidence of dogs undergoing limb amputation post-RT, though all dogs that underwent post-treatment amputation had received RT (2/12 SBRT, 2/12 PRT) for primary tumor treatment. Importantly, the vast majority of dogs had very good function of the limb following partial ulnar ostectomy without any need for surgical stabilization of the carpus or elbow. Postoperative external coaptation was common, but variable with regards to use of splint vs. soft padded, and largely dependent on clinician preferences. Regardless, the majority of dogs were reported to have good limb function following ulnectomy.

The majority of dogs with distal ulnar tumors underwent partial ulnectomy (14/23, 60.9%) as a limb preserving local treatment option rather than SBRT (6/23, 26.1%) or PRT (3/23, 13%), likely owing to the good expected function with distal ulnectomy as the distal ulna is responsible for minimal weight bearing (15). Although two cadaveric studies have demonstrated carpal laxity following excision of the lateral styloid process (and concurrently lateral collateral ligament), the 8 dogs with lateral styloid process excision in this study retained good function long-term postoperatively, with only 1/8 (12.5%) dog having transient carpal instability that did not require surgical stabilization (aside from soft tissue imbrication during closure) or surgical revision (10, 11). Therefore, based on the results of this study, dogs that undergo lateral styloid process excision during partial ulnectomy can be expected to have very good clinical function without the need for surgical stabilization or long-term external coaptation.

In addition, many dogs with mid ulnar tumors also underwent partial ulnectomy with good outcomes, but no dogs with proximal ulnar tumors underwent partial ulnectomy. This is likely owing to the important anatomical structures of the proximal ulna that are involved in weight bearing and elbow function and cannot be readily excised (16). Similarly, no dogs with possible tumor invasion of the elbow joint or osseous lesions other than the antebrachium underwent partial ulnectomy, likely due to the potential for residual gross disease with partial ulnectomy alone. Importantly, however, multiple dogs had radial changes on imaging and underwent partial ulnectomy with good outcomes and no evidence of local recurrence. This finding highlights the importance of considering differentials of local reaction and tumor invasion for changes of the adjacent radial bone. Radial imaging changes may not be a contraindication for surgery, though this decision needs to be made in light of the extent of radial changes and relative ranking of the differential diagnoses (tumor invasion vs. benign change) for a given case.

This study provides important functional information on dogs that undergo partial ulnectomy with interosseous ligament transection. The interosseous ligament was transected completely in 4 dogs that each underwent concurrent radius-ulna stabilization, and it was partially transected in 4 dogs that did not undergo any surgical radius-ulna stabilization. All of these dogs retained adequate limb function long-term, though 2 of the dogs with radius-ulna stabilization experienced biomechanical complications associated with failure of this repair (hemicerclage breakage). Although theoretically with transection of the interosseous ligament, stabilization of the proximal ulna to radius is needed to prevent distraction of the proximal ulna or radial head luxation, this stabilization of the proximal ulna to radius failed in 2/4 dogs with complete transection of the interosseous ligament within approximately 1 month postoperatively, and neither dog required surgical revision though 1 dog remained moderately lame long-term (the other dog was weight bearing normally on long-term follow-up). Therefore, the clinical significance of radius-ulna stabilization following complete transection of the interosseous ligament with partial ulnar ostectomy is not currently known based on these results, and additional studies are needed.

Overall, partial ulnectomy, SBRT, and PRT were well tolerated with a low incidence of clinically important surgical or RT complications in each group. Although grade 1 and 2 complications occurred in many surgical patients, no higher grade complications occurred intra- or post-operatively for any dog that underwent partial ulnectomy. Also, all dogs tolerated PRT very well with no acute or late RT-associated complications. Though SBRT was well tolerated in the majority of dogs that received this treatment, 4 dogs did develop high grade (grade 3) acute (3/12, 25%) and late (2/12, 16.7%) complications of the skin (1 dog had grade 3 acute and late complications), with one complication ultimately resulting in death. However, it is also important to note that 2 dogs that developed grade 3 acute complications following SBRT had other confounding factors (such as concurrent surgery for radial fracture repair and a pre-existing wound) that may have contributed to these complications, and the effect that SBRT alone had in development of these complications is unclear. In addition, a previous study demonstrated that treatment of bone tumors with SBRT and surgical stabilization is associated with a high rate of complications, and as such these procedures are no longer performed together at our institution (17). Furthermore, based on the outcomes of cases in this study, at our institution we no longer consider patients with wounds in the region of their tumors to be appropriate candidates for SBRT, and our protocol for skin constraints has been modified as recently documented in an effort to reduce severe skin-associated SBRT effects (9).

Moreover, of dogs that underwent RT for primary local treatment of their ulnar tumors, pathologic fractures (4/12 [33.3%] for SBRT, 5/12 [41.7%] for PRT), local tumor progression (5/12 [41.7%] for SBRT, 2/12 [16.7%] for PRT), and/or subsequent surgery of the limb (4/12 [33.3%] for SBRT, 2/12 [16.7%] for PRT) occurred in 58.3% (7/12 [58.3%] for both SBRT and PRT). Alternatively, for dogs that underwent partial ulnectomy for primary treatment of their ulnar tumors, none developed fractures, local tumor recurrence was relatively uncommon (2/16, 12.5%), and none underwent subsequent surgery of the limb. However, the only significant difference detected with regards to these outcomes was the incidence of pathologic fracture between ulnectomy and PRT patients, and the overall functional and primary tumor-associated outcomes were good for dogs that underwent ulnectomy, SBRT, and PRT.

This study had several limitations. First, the study was retrospective in nature with incomplete clinical information and loss to follow-up for some patients. This may have resulted in under- or over-interpretation of findings, including documentation of functional and disease progression outcomes. Similarly, due to the retrospective nature of data collection, a variety of local imaging and staging diagnostics were used based on clinical findings and client decisions, which may have influenced the findings of this study. Importantly, because of the relatively small sample size of dogs in each treatment subgroup (partial ulnectomy, SBRT, PRT), statistical power was limited to detecting only the largest differences, and greater sample sizes could result in more statistically significant findings. Also, due to the retrospective nature of data collection, it was not possible to statistically assess the influence of factors such as primary tumor size, percentage of ulna affected, or soft tissue expansion of the tumor relative to survival outcomes or treatment modality selected. It is possible that multiple RT cases included dogs with more locally advanced disease or tumors that were not readily amenable to excision, which was not possible to capture in our retrospective analysis and may have influenced the outcome data. Another limitation involves the various tumor types, as inclusion required an ulnar tumor without any specific cytological or histopathological diagnosis of a certain tumor type. Thus, this population represents patients with a variety of diseases, and not solely osteosarcoma, though osteosarcoma was the most common diagnosis in this population. Finally, based on inclusion criteria of this study, no comments can be made on partial ulnar ostectomy or RT as limb preserving local treatment strategies in comparison to full limb amputation or other local treatments. Additional studies are needed to evaluate larger numbers of dogs undergoing these primary treatment modalities, and it is possible that dogs undergoing each treatment type may represent different populations relative to tumor location/extent and metastatic disease burden.

In conclusion, this study represents the largest report to date on dogs with ulnar bone tumors that underwent local limb sparing treatment *via* partial ulnar ostectomy, SBRT, or PRT. This information can be used to inform clinicians and owners about these treatment options, complications, functional outcomes, and disease progression outcomes. Overall, the survival data presented in this study are similar to those found in canine primary bone tumor literature, and our findings support the use of adjuvant chemotherapy in conjunction with local treatment (surgery or RT) for prolongation of survival times. For dogs undergoing definitive therapy (characterized by primary treatment and chemotherapy) in this study, partial ulnectomy resulted in significantly improved survival times relative to SBRT. However, owner goals, patient comorbidities, and extent of disease are important factors for individual treatment decisions, and results of this study support that good outcomes can occur with partial ulnectomy, SBRT, or PRT for local limb preserving treatment of ulnar tumors in dogs. For owners that are risk-averse to invasive therapies or seeking palliative treatment options in the face of an overall poor prognosis with bone tumors in dogs, RT options for local disease control and palliation can provide good functional outcomes with a low incidence of major complications. Ultimately, this data supports that dogs undergoing partial ulnectomy or RT for treatment of ulnar tumors overall have good long-term function of the limb without any need for additional surgical stabilization of the carpus and survival times similar to previously reported for dogs with appendicular bone tumors.

## Data availability statement

The original contributions presented in the study are included in the article/Supplementary material. Further inquiries can be directed to the corresponding author.

## Ethics statement

Ethical review and approval was not required for the animal study because this is a retrospective study reporting on outcomes of patients (with an uncommon disease) that were treated according to standard of care practices as elected by owners, and not in a clinical trial. Permission to use medical records of patients for publication is obtained *via* client consent by our hospital at admission. Written informed consent was obtained from the owners for the participation of their animals in this study.

## Author contributions

MG, TM, DT, and DW participated in the data acquisition and manuscript preparation. DT provided the statistical analysis for this study. All authors contributed to the article and approved the submitted version.

## Conflict of interest

The authors declare that the research was conducted in the absence of any commercial or financial relationships that could be construed as a potential conflict of interest.

## Publisher’s note

All claims expressed in this article are solely those of the authors and do not necessarily represent those of their affiliated organizations, or those of the publisher, the editors and the reviewers. Any product that may be evaluated in this article, or claim that may be made by its manufacturer, is not guaranteed or endorsed by the publisher.
